# Bidirectional Ventricular Tachycardia Secondary to Dichloromethane Intoxication: A Case Report

**DOI:** 10.7759/cureus.101009

**Published:** 2026-01-07

**Authors:** Luís Henrique Sardinha Borborema, Daniel A Adedd Filho

**Affiliations:** 1 Internal Medicine, Hospital das Clínicas da Faculdade de Medicina da USP, São Paulo, BRA; 2 Emergency Department, Hospital Universitário da Faculdade de Medicina da USP, São Paulo, BRA

**Keywords:** bidirectional ventricular tachycardia, dichloromethane, halogenated hydrocarbons, ventricular arrhythmia, volatile inhalant abuse

## Abstract

Bidirectional ventricular tachycardia (BVT) is a rare ventricular tachyarrhythmia, and only a limited number of etiologies have been described to date. We report the case of a previously healthy 30-year-old woman who developed BVT followed by cardiac arrest after accidental ingestion of dichloromethane, an organochlorine solvent used in illicit volatile inhalant preparations. Further evaluation revealed no structural heart disease, electrolyte disturbance, or exposure to medications classically associated with BVT, supporting dichloromethane intoxication as a plausible and, to our knowledge, previously unreported trigger of this arrhythmia.

## Introduction

BVT is a rare ventricular tachyarrhythmia marked by beat-to-beat alternation in QRS axis and morphology on the frontal plane [1]. Accurately diagnosing this distinctive electrocardiogram (ECG) pattern is clinically significant because it can prevent the development of potentially reversible and high-risk conditions, such as ventricular fibrillation and cardiac arrest [1]. Historically, it was first described by Schwensen et al. in 1922 and associated with digitalis toxicity [2]. Although it remains the most common cause of BVT, other conditions have also been recognized as triggers, such as catecholaminergic polymorphic ventricular tachycardia (CPVT), acute myocardial ischemia, cardiac sarcoidosis, and drug overdose with caffeine and aconitine [1,3,4]. Here, we describe a case of BVT secondary to acute intoxication with an illicit inhalant primarily composed of dichloromethane. Since organochlorine compounds are commercially available as industrial chemical solvents, their misuse as inhaled illicit drugs has been reported in some countries [5]. Exposure to these agents has been associated with severe toxicity, including a substantial risk of sudden death or long-term health sequelae [6,7]. To our knowledge, this is the first report of BVT associated with exogenous intoxication from recreational inhalants containing organochlorine compounds.

## Case presentation

A 30-year-old woman was admitted to the emergency department with an altered level of consciousness. The accompanying relative reported that she had used a volatile recreational inhalant and consumed alcoholic beverages but had not used any other illicit drugs. She had accidentally ingested the inhalant drug approximately 30 minutes before arrival at the hospital, leading to a rapid loss of consciousness. She was not taking any regular medications, had no significant past medical history, and had no relevant family history, including sudden cardiac death.

**Figure 1:**
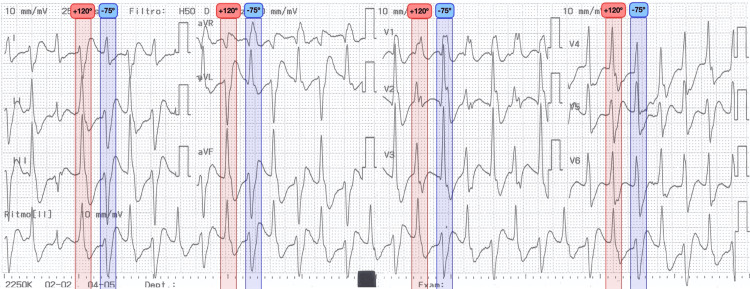
Bidirectional ventricular tachycardia with alternating QRS axis Twelve-lead electrocardiogram showing bidirectional ventricular tachycardia with a ventricular QRS morphology alternating between two frontal plane axes, +120° and −75°).

**Figure 2:**
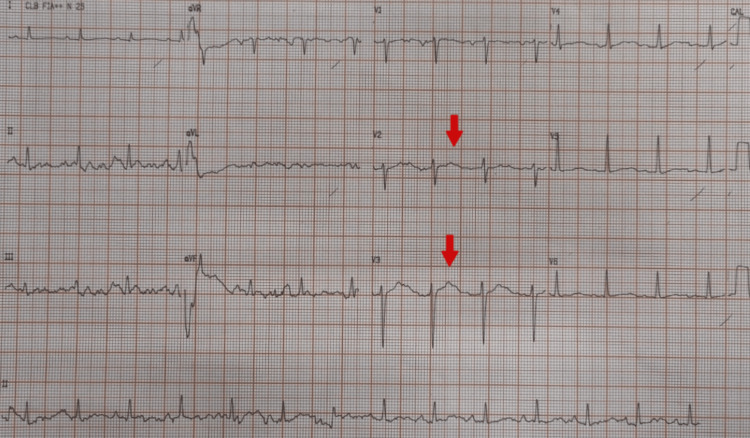
Post-return of spontaneous circulation (post-ROSC) ECG A 12-lead electrocardiogram obtained after return of spontaneous circulation demonstrated sinus rhythm with a prolonged corrected QT interval, most clearly seen in leads V2 and V3. ROSC: Return of Spontaneous Circulation, ECG: Electrocardiogram

Physical examination showed no eye opening, no verbal response, abnormal flexion of the upper limbs, conjugate lateral gaze deviation, abnormal tonic flexion of the left upper limb, and sialorrhea, with otherwise normal vital signs. Given this presentation, an epileptic seizure was suspected, and intravenous phenytoin was initiated at a dose of 20 mg/kg and an infusion rate of 35 mg/min. Approximately five minutes after the infusion was started, she developed a hemodynamically stable wide QRS complex tachycardia. The 12-lead electrocardiogram showed a ventricular tachycardia with alternating frontal plane QRS axis (+120° and −75°) and atrioventricular dissociation, consistent with BVT (Figure 1). The phenytoin infusion was discontinued, but the rhythm degenerated after 3 minutes to a ventricular fibrillation cardiac arrest. Cardiopulmonary resuscitation was carried out for 25 minutes, resulting in the return of spontaneous circulation in sinus rhythm (Figure 2) with a QRS duration of <120 ms and a markedly prolonged QT interval. After stabilization, she was transferred to the intensive care unit (ICU). Laboratory tests demonstrated metabolic acidosis on arterial blood gas analysis, without significant electrolyte abnormalities (Table 1).

**Table 1:** Arterial blood gas and key serum electrolytes on presentation

Test	Patient result	Reference range
Arterial pH	7.25	7.35 – 7.45
PaCO₂	37 mmHg	35 – 45 mmHg
Bicarbonate (HCO₃⁻)	16 mmol/L	22 – 28 mmol/L
Base excess	−10.4 mmol/L	−2 to +2 mmol/L
Lactate	41.6 mg/dL	4.5 – 19.8 mg/dL
Anion gap	15	8 – 16
Potassium (K⁺)	3.6 mEq/L	3.5 – 5.0 mEq/L
Ionized calcium (Ca²⁺)	1.15 mmol/L	1.12 – 1.32 mmol/L
Magnesium (Mg²⁺)	1.8 mg/dL	1.7 – 2.2 mg/dL
Sodium (Na⁺)	135 mEq/L	135 – 145 mEq/L

Throughout the hospitalization, the patient had no further arrhythmic episodes and no neurological symptoms. On admission day, a transthoracic echocardiogram and cranial computed tomography were obtained, both without significant abnormalities. On hospital day one, due to worsening abdominal pain, an upper gastrointestinal endoscopy was performed, which revealed extensive necrosis of the esophageal and gastric walls. On hospital day three, she developed signs of an acute abdomen concerning for perforation and underwent emergent total gastrectomy and partial esophagectomy. Reconstruction of the gastrointestinal tract was later performed as an elective procedure. She remained hospitalized for three weeks and was discharged without cardiac or neurological sequelae.

**Figure 3:**
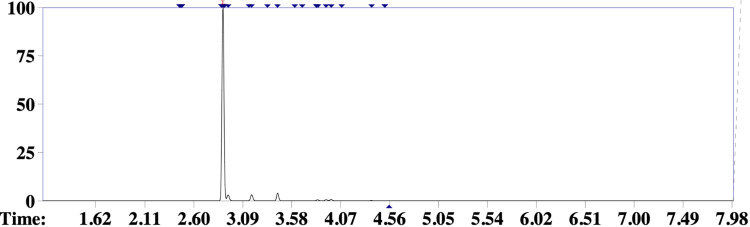
Gas chromatogram of the inhalant sample Gas chromatogram of the inhalant drug sample, demonstrating a peak with a retention time of 2.894 s, consistent with dichloromethane.

**Figure 4:**
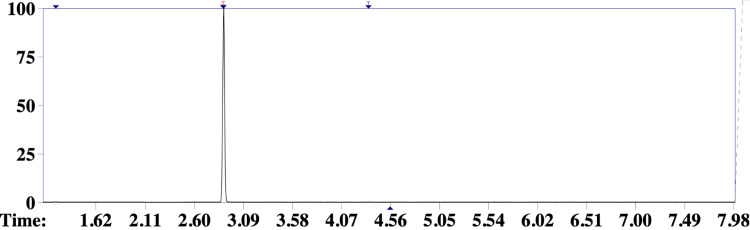
Gas chromatogram of the reference dichloromethane sample Gas chromatogram of a reference dichloromethane sample demonstrating a retention time of 2.894 s, matching the main peak identified in all samples.

**Figure 5:**
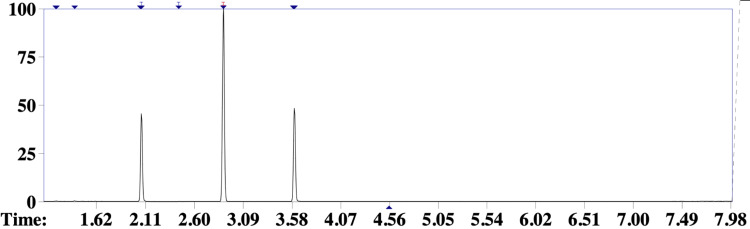
Gas chromatogram of the patient’s blood sample Gas chromatogram of the patient’s blood sample showing three peaks at different retention times, corresponding to ethyl alcohol (2.069 s), dichloromethane (2.894 s), and n-propanol (3.604 s), the latter used as an internal control.

Subsequently, samples of the inhalant preparation and the patient’s blood were obtained for toxicological analysis. In order to identify the components of the volatile mixture, headspace gas chromatography coupled to mass spectrometry (HS-GC-MS) was performed at the local police toxicology laboratory [8]. The main volatile component detected in the ingested drug sample was compatible with dichloromethane (Figures 3, 4), an industrial solvent belonging to the organochlorine class and frequently found in inhaled illicit substances [5]. In addition, analysis of the patient’s blood confirmed the presence of the same compound, as well as ethanol (Figure 5).

## Discussion

BVT is a rare subtype of ventricular tachycardia characterized by a distinctive electrocardiographic pattern with beat-to-beat alternation in QRS axis and morphology on the frontal plane [9]. The most common morphology for BVT in the literature is a right bundle branch with alternating axis [3]. Other described patterns include beat-to-beat alternation between left and right bundle branch block morphologies [10], as well as narrow QRS complex tachycardia with an alternating frontal plane axis [11], as observed in our patient. Several mechanisms have been proposed to explain the development of BVT. One of them, known as the “ping-pong” theory, involves abnormal automaticity in which a ventricular extrasystole originating in the Purkinje fibers triggers another extrasystole within the His system, thereby establishing the characteristic alternating pattern of BVT [9]. Reentry has also been described as a mechanism initiating BVT. Ueda-Tatsumoto et al. reported a patient with arrhythmogenic right ventricular cardiomyopathy in whom a macroreentrant circuit with a common critical isthmus utilized two different exit sites, resulting in BVT [12]. Bidirectional ventricular tachycardia may further degenerate into polymorphic ventricular tachycardia or ventricular fibrillation if additional foci of depolarization or reentrant circuits arise [1].

One of the triggers for these arrhythmias is intoxication, including toxicity from digoxin, caffeine, or aconitine [1,2,4]. We report a patient with inhalant exposure, an underrecognized form of intoxication characterized by the misuse of readily available commercial products (e.g., nail polish remover, glue, or spray paints) as psychoactive substances [13]. Over the past decade, organochlorine compounds such as dichloromethane have been increasingly identified in illicit inhalant preparations [5]. These agents are widely available because they are used in a range of industrial processes, primarily as lipophilic solvents [5]. Although the recreational use of volatile substances has declined over the past two decades, it remains an important public health problem. In 2024, 10.2% of 8th-grade adolescents (13-14 years) and 5.3% of 12th-grade adolescents (17-18 years) reported lifetime inhalant use in the United States [14]. 

Intoxication with these substances is associated with a substantial risk of death. Several fatalities related to volatile substance use have been reported in the literature [6, 7], although the true burden is likely higher than suggested because these events are often unrecognized, undiagnosed, or misclassified [15]. In the acute setting, death may result from hypoxia, cardiac dysfunction, or a depressed level of consciousness [6]. In our case, the patient initially presented with a seizure-like episode and subsequently developed a BVT, followed by ventricular fibrillation cardiac arrest. Beyond these nonspecific mechanisms of acute toxicity, exposure to halogenated hydrocarbons such as dichloromethane has been associated with arrhythmias attributed to increased cardiac sensitivity to catecholamines, although the underlying mechanisms remain poorly understood [16, 17]. These compounds may also produce dose-dependent inhibition of cardiac potassium channels in cardiomyocytes, particularly hERG (I_Kr) and I_Ks [18]. Inhibition of these channels can alter action potential morphology and impulse conduction, leading to arrhythmogenesis, including torsade de pointes [19]. At higher concentrations, halogenated hydrocarbons may additionally inhibit sodium channels, slowing conduction velocity and altering the refractory period, which may promote reentrant arrhythmias, especially in the presence of catecholamines [16].

In light of these mechanisms, it is plausible that ingestion of the dichloromethane-based inhalant contributed to cardiotoxicity through mechanisms similar to those described for other halogenated hydrocarbons and, thereby, played a central role in the arrhythmia observed in this case. The detection of dichloromethane in both the patient’s blood and the inhalant sample strongly supports an association between exposure to this substance and the episode of BVT described. This interpretation is further supported by the absence of other potentially confounding drugs apart from ethanol and by the lack of recurrent arrhythmias during hospitalization. Further studies are needed to better define the contribution and true prevalence of ventricular tachyarrhythmias in cases related to volatile inhalant use and to elucidate the mechanisms by which these agents may precipitate BVT.

## Conclusions

We presented a case of bidirectional ventricular tachycardia complicated by cardiac arrest secondary to accidental exposure to an organochlorine solvent. Further evaluation did not identify structural heart disease, electrolyte abnormalities, or medication exposures classically associated with this rare ECG pattern, supporting dichloromethane intoxication as a plausible precipitant and potentially expanding the differential diagnosis for this type of arrhythmia. This case underscores the potential for severe cardiotoxicity from volatile solvents and highlights the importance of considering toxic exposures when BVT is encountered. Further studies are needed to clarify causality and to better characterize the mechanisms by which such exposures may precipitate ventricular tachyarrhythmias.
